# Generation and selection of pluripotent stem cells for robust differentiation to insulin-secreting cells capable of reversing diabetes in rodents

**DOI:** 10.1371/journal.pone.0203126

**Published:** 2018-09-05

**Authors:** Sheryl M. Southard, Rama P. Kotipatruni, William L. Rust

**Affiliations:** SERAXIS Inc, Germantown, Maryland, United States of America; University of Kansas Medical Center, UNITED STATES

## Abstract

Induced pluripotent stem cell (iPSC) technology enables the creation and selection of pluripotent cells with specific genetic traits. This report describes a pluripotent cell line created specifically to form replacement pancreatic cells as a therapy for insulin-dependent diabetes. Beginning with primary pancreatic tissue acquired through organ donation, cells were isolated, re-programmed using non-integrating vectors and exposed to a four day differentiation protocol to generate definitive endoderm, a developmental precursor to pancreas. The best performing iPSC lines were then subjected to a 12-day basic differentiation protocol to generate endocrine pancreas precursors. The line that most consistently generated highly pure populations was selected for further development. This approach created an iPSC-variant cell line, SR1423, with a genetic profile correlated with preferential differentiation toward endodermal lineage at the loss of mesodermal potential. This report further describes an improved differentiation protocol that, coupled with SR1423, generated populations of greater than 60% insulin-expressing cells that secrete insulin in response to glucose and are capable of reversing diabetes in rodents. Created and banked following cGMP guidelines, SR1423 is a candidate cell line for the production of insulin-producing cells useful for the treatment of diabetes.

## Introduction

Insulin-dependent diabetes can be controlled by replacement cell therapy. In the clinic this is accomplished by transplant of allogeneic donor pancreatic islets of Langerhans in conjunction with anti-rejection immune suppression [1–3]. This strategy has been improved in animal models by generating insulin-producing (beta) cells from human stem cells, and transplanting those within devices that obviate the need for immune suppression [4,5]. If made practical and efficacious for human patients, such a strategy would revolutionize treatment for a currently incurable disease that is reaching global, epidemic proportions.

Human embryonic stem cells (hESC) and induced pluripotent stem cells (iPSC) are proven sources of surrogate beta cells for a potential replacement cell therapy [6–8]. To achieve this, hESC and iPSC are guided along developmental pathways in vitro to produce cells with hallmarks of bona fide pancreatic beta cells and which secrete insulin in response to glucose in the cell culture media [8,9].

Previous studies have shown that pluripotent cell lines can vary widely in their ability to differentiate to certain lineages [10–13]. Furthermore, protocols established to guide stem cell differentiation towards the beta cell phenotype also vary widely [8,9,14,15]. Each of these protocols was optimized using a specific stem cell line. Collectively, we interpret this to imply that each pluripotent cell line requires a unique protocol to achieve the most robust result.

In an effort to create an iPSC line for use as a cell replacement therapy for diabetes, our group developed a line that consistently and robustly differentiates to beta cells pursuant to a relatively simple, defined, and xeno-free differentiation protocol [16]. We began with primary pancreatic donor tissue based on reports that residual epigenetic patterning could enhance the likelihood of reprogramming a cell line with a high tendency to differentiate back to the pancreatic lineage [17,18]. We chose a simple method using small-molecules and xeno-free reagents to facilitate clinical translation of the final therapeutic candidate.

The concept of creating a cell line to respond to a protocol rather than creating a protocol to control a cell line is a simple strategy for improved efficiency that is rarely used in the field. The selected cell line, SR1423, differentiates preferentially to endodermal tissue compared to mesodermal tissue, and is capable of generating highly pure populations of pancreatic and insulin-producing cells. Gene expression analysis shows that SR1423 has a genetic signature that correlates with the ability to respond to a basic pancreatic differentiation protocol. In anticipation of translation to the clinic, SR1423 was derived, expanded and banked following good manufacturing practice (cGMP) guidelines.

We next endeavored to optimize our differentiation protocol to maximize the output of insulin-producing cells using SR1423. A unique feature of this protocol is the elimination of steps and reagents commonly used in other leading protocols. With this amended protocol, we were able to achieve cultures with high insulin production. SR1423 cells differentiated to the insulin-secreting phenotype are capable of rescuing hyperglycemia in animal models.

## Materials and methods

### Islet harvest

Properly consented and anonymized whole human pancreata were obtained from registered organ donation. The lobes were injected with collagenase P (Roche #1129 002 001) re-suspended to 1.4 mg/ml in islet isolation solution (Hanks Balanced Salt Solution (Invitrogen #14065–056) containing 0.35g NaHCO3/L and 1% Human Serum Albumin (Roche A9731)). The inflated lobes were incubated at 37°C for 15–25 minutes with mild agitation. The digest was diluted with cold islet isolation solution and centrifuged at 1500RPM for 5 minutes. The supernatant was discarded and the pellet was washed in cold islet isolation solution with vigorous trituration. The solution was filtered through a 420μm sieve (Bellco Glass, Inc #1985–00040) and centrifuged. The pellet was resuspended in 1.100 g/ml Histopaque (Sigma #10771, Sigma #11191) and centrifuged for 30min at 1200RPM. The supernatant was collected, diluted 2X in islet isolation solution and centrifuged at 1500RPM for 5 min. The pellet was rinsed in islet isolation solution, centrifuged, and cultured in E8 medium (Gibco #A1517001) in a humidified incubator at 37°C and 5% CO_2_. The following day, the islets were centrifuged at 1500 RPM for 5 minutes and re-suspended in undiluted TryplE Select 10X (Life Technologies #A12177) and incubated for 10 minutes at 37°C. The dissociated islets were diluted in E8 media, centrifuged, re-suspended in E8 supplemented with 100 ng/ml hydrocortisone (Sigma #H0135), 1U/ml thrombin (Sigma #T9326), and 100 ng/ml EGF (Sigma E5036). Cells were cultured on dishes coated with vitronectin (Life Technologies #A14700) following manufacturer’s instructions.

### Reprogramming

Cells were rinsed with PBS (Gibco #14190144) and incubated in TryplE select 1X for 5 minutes at 37°C. Digestion was arrested with E8 medium and cells centrifuged at 1000RPM for 5 minutes. Cells were re-suspended in BTX electroporation solution (VWR #89130–542) at 2 x 10^6^ cells/200 μl and added to electroporation cuvette with 20ug of reprogramming plasmids. 2 reprogramming plasmids comprising EBNA episomal expression sequences, ampicillin resistance, and the reprogramming genes OCT4, SOX2, KLF4, and L-MYC under the control of the CMV promoter were constructed in-house. Electroporation cuvette was pulsed using a gene pulser Xcell (Bio-Rad). Cells were transferred to vitronectin-coated dishes in E6 medium (Life Technologies #a1516401) supplemented with 100 ng/ml bFGF (Life Technologies #PHG6015) and 1 μM hydrocortisone. Cells were cultured at 37°C in a humidified incubator with 5% CO_2_. After 24 hours, media was changed with E6 supplemented with 100 ng/ml bFGF, and 1 μM hydrocortisone, and 100 μM sodium butyrate (Sigma #P1269) and changed every other day. Stem cell colonies were manually detached and transferred to vitronectin-coated dishes in E8 medium. It is unknown whether the reprogrammed cells originated from beta, alpha, PP, grehlin, or other cells associated with islets. Primary cell culture and reprogramming occurred in a monitored cleanroom following cGMP guidelines.

### Stem cell culture

Undifferentiated iPS cells were maintained in 6-well tissue culture plates (Greiner Bio-One #657160) coated with vitronectin XF (Stem Cell Technologies #07180) or 17 μg/cm^2^ Geltrex (Life Technologies #A1413301) following manufacturer’s instructions and fed daily with E8 medium. Cultures were passaged at 75–85% confluence every 3–5 days with 0.5 mM EDTA (Life Technologies #15575) and seeded at 7 x 10^3^ cells/cm^2^.

### Stem cell selection

Undifferentiated iPS cells were seeded at 6.3 x 10^4^ on 6-well tissue culture plates as described under section 2.3. 24–48 hours after plating, adherent cells were rinsed with dPBS (-Mg^2+^/-Ca^2+^) and cultured in DMEM/F-12 medium (Life Technologies #10565018), 0.2% HSA, 1XB27 supplement (Life Technologies #A1486701), 100 ng/ml Activin A (PeproTech #AF-120-14E) and 1 μM Wortmannin (Sigma #W3144) with daily media changes for four days. On the fourth day, cultures were fixed and visualized by immunocytochemistry for expression of the endodermal markers HNF3beta and SOX17 as described in sections 2.7 and 2.8. Cell lines with near-uniform co-expression of these markers by visual inspection were selected for secondary selection. See Supplemental S1 Fig for examples.

Secondary selection was performed by seeding undifferentiated iPS cells at 6.3 x 10^4^ on 6-well tissue culture plates as described under section 2.3. 24–48 hours after plating, adherent cells were rinsed with dPBS (-Mg^2+^/-Ca^2+^) and cultured in DMEM/F-12 medium (Life Technologies #10565018), 0.2% HSA, 1XB27 supplement (Life Technologies #A1486701), 100 ng/ml Activin A (PeproTech #AF-120-14E) and 1 μM Wortmannin (Sigma #W3144) with daily media changes for four days. On days 5,6,7, and 8 cells were cultured in DMEM (Life Technologies #10567014), 0.2% HSA, 1XB27 supplement, 4μM Retinoic Acid (Sigma #R2625), 50 ng/ml KGF (PeproTech #AF-100-19), 50 ng/ml Noggin (PeproTech #120-10C), 0.25 μM Cyclopamine KAAD (Millipore #239804). On days 9 and 11 medium was changed with DMEM, 0.2% HSA, 1XB27 supplement, 50 ng/ml KGF, 50 ng/ml Noggin, 50 ng/ml EGF (PeproTech #AF-100-15); On the thirteenth day, cultures were fixed and visualized by immunocytochemistry for expression of the pancreatic markers Pdx1 and Nkx6.1 as described in sections 2.7 and 2.8. Cell line #1423 consistently displayed high co-expression of these markers and was selected for further characterization and downstream experiments. See Supplemental S2 Fig for examples.

### Differentiation

SR1423 cells or BGO1V cells (MTI-Globalstem #GSC-1103) were seeded at 6.3 x 10^4^ as described above and allowed to grow for 18–24 hours. Cells were then washed with dPBS (-Mg^2+^/-Ca^2+^) and medium was changed following a 28 day schedule comprised of 6 media formulation as follows: Days 1, 2, 3: DMEM/F-12 medium (Life Technologies #10565018), 0.2% HSA, 1XB27 supplement (Life Technologies #A1486701), 100 ng/ml Activin A (PeproTech #AF-120-14E) and 1 μM Wortmannin (Sigma #W3144); Days 4, 5, 6, 7: DMEM (Life Technologies #10567014), 0.2% HSA, 1XB27 supplement, 4 μM Retinoic Acid (Sigma #R2625), 50 ng/ml KGF (PeproTech #AF-100-19), 50 ng/ml Noggin (PeproTech #120-10C), 0.25 μM Cyclopamine KAAD (Millipore #239804); Days 8, 10: DMEM, 0.2% HSA, 1XB27 supplement, 50 ng/ml KGF, 50 ng/ml Noggin, 50 ng/ml EGF (PeproTech #AF-100-15); Days 12, 14: DMEM, 0.2% HSA, 1XB27 supplement, 50 ng/ml Noggin, 50 ng/ml EGF, 1 μM γ-secretase inhibitor XXI (Millipore #565790), 10 μM Alk5i II (Axxora, #ALX-270-445); Days 16, 18: DMEM, 0.2% HSA, 1XB27 supplement, 10 μM Alk5i II, 100 nM Retinoic Acid; Days 20, 22, 24, 26, 28: CMRL (Life Technologies #11530037), 0.2% HSA, 1X B27 supplement, 1X glutamax (Life Technologies # 35050061), 10 μM Alk5i II, 10 mM Nicotinamide (Sigma #N0636), 50 ng/ml IGF-I (PeproTech #100–11), 10 ng/ml BMP4 (PeproTech #120-05ET). For differentiations with the addition of a second stage employing KGF, medium on days 4, 5, 6 with DMEM, 0.2% BSA, 1XB27 supplement, and 50 ng/ml KGF was inserted into the schedule shifting the remaining stages three days later. T3 (1μM, Sigma #T6397) was employed on days 12–28 for samples used for flow cytometry analysis and for immunocytochemical analysis of glucagon, C-peptide and NeuroD1 expression. For germ layer differentiation, the Human Pluripotent Stem Cell Functional Identification Kit (R&D systems #SC027B) was used following manufacturer’s instructions.

### Glucose-stimulated insulin secretion

Cells were pre-incubated in 2mM glucose in KREBs for 30–60 minutes prior to exposure to glucose solutions. Cells were incubated in 2 mM glucose in KREBS (Alfa Aesar # J67591-AP) for 30 minutes and supernatant collected. Buffer was changed for 20 mM glucose in KREBS for 30 minutes and supernatant collected. Buffer was changed for 20 mM glucose, 30 mM KCl in KREBS for 30 minutes and supernatant collected. The concentration of C-peptide in each supernatant was determined using an Ultrasensitive C-peptide ELISA (Mercodia #10-1141-01), for Glucagon using Glucagon ELISA (Mercodia #10-1271-01) and a GENios microplate reader (TECAN). Absorbance readings were measured in duplicate using Magellan software (TECAN).

### Immunocytochemistry

Cells were fixed with 4% paraformaldehyde in dPBS for 20 minutes, permeabilized with 0.3% Triton X-100 in dPBS for 15 minutes and blocked with 0.1% Triton X-100 (VWR #97062–208) and 2.5% horse serum albumin in dPBS for 1 hour. Incubation with primary antibodies was performed overnight at 4°C, and secondary antibodies were incubated the following day for 2 hours in the dark. Nuclei were then stained with Hoechst solution (Sigma Aldrich #H6024) for 20 minutes. Cells were washed with dPBS between each step. All images were taken with a Motic AE30/31 Epi-fluorescence microscope. For generating clear images of late-stage planar cultures, cells were scraped off the tissue culture surface and rotating at 95RPM in six-well suspension culture dishes in differentiation media supplemented with Y27632 (Biogems #1293823). The resultant clusters were fixed with 4% paraformaldehyde in dPBS for 20 minutes, rinsed, and allowed to gravity settle in a solution of 30% sucrose before embedding and cryopreserving in Optimum Cutting Temperature medium (VWR # 95057–838). Cryotome sections were placed on silane-coated microscope slides and processed as with planar cultures.

### Antibodies

Primary antibodies used: Pdx1 (1:500, R&D Systems # AF2419 [AB_355257]), Nkx6.1 (1:50, DSHB #F55A12 [AB_532579]), C-peptide (1:100, DSHB #GN-ID4 [AB_2255626]), Insulin (1:200, Cell Signaling #3014S [AB_2126503]), Glucagon (1:100, Cell Signaling Technology #2760S [AB_10698611]), NeuroD1 (1:500, Abcam #ab60704 [AB_943491]), Sox17 (1:200, R&D Systems # MAB19241 [AB_10920934]), HNF3Beta (1:200, Cell Signaling, #8186S [AB_10892612]) and ESC differentiation kit (Applied Stem Cell # ASK-3006). Secondary antibodies: rabbit anti-goat 555 (1:1000, Thermo Fisher Scientific # A21431 [AB_2535852]), anti-mouse Dylight 488 (1:1000m, Vector # DI-2488 [AB_2307439]), anti-rabbit Dylight 488 (1:1000, Vector #DI-1088 [AB_2336403]). In flow cytometry experiments, conjugated antibodies were employed as follows: PerCP-Cy5.5 mouse anti-Pdx1 (1:20, BD Biosciences #563436), Alexa Fluor 647 mouse anti-Nkx6.1 (1:20, BD Biosciences #563338), PE mouse anti-Glucagon (1:20, BD Biosciences #565860), Alexa Fluor 488 mouse anti-ProInsulin (1:50, R&D Systems # IC13361G)

### Gene analysis

Undifferentiated SR1423 cultures were dissociated with 0.5 mM EDTA in dPBS, washed with ice-cold dPBS, and flash frozen with liquid nitrogen. Samples were stored at -80 °C until RNA was isolated with the RNeasy Mini Kit (Qiagen #74104). Isolates were treated with TURBO DNase (Ambion #AM2238) and RNA clean-up was performed on a second RNeasy Mini Kit column immediately following isolation. Purity and yield of RNA was confirmed using a NanoDrop 2000c spectrophotometer (ThermoFisher Scientific) and isolated RNA was stored at -80°C until RT-qPCR was performed. Reverse transcription to cDNA was performed using the iScript cDNA Synthesis Kit (BioRad #1708890) and a C1000 thermal cycler (BioRad). qPCR was performed using 50 ng cDNA in the SsoAdvanced Universal SYBR Green Supermix (BioRad #1725270), and a CFX96 real-time PCR system paired with a C1000 thermal cycler (BioRad). All experimental samples were measured in triplicate and the experiment was performed twice. All primers were added at 400 nM and starting cDNA quantity was normalized across reactions with the housekeeping gene GAPDH. All sequences of primers (Sigma Aldrich) were listed in Table 1.

**Table 1:** PCR primers used in this study.

Gene Symbol	Forward	Reverse
BHMT2	AAGTGGGAAGATGTAAAT	GCTTCATCCTTCTGGTAT
COX7A1	AACAGAAGCTCTTCCAGG	CAGGCTTCTTGGTCTTAA
HSPB2	ATGATGGCATCTTAAACC	AGATGTAGACCTCATTGA
NAP1L1	ATTATGGGTTGTACAGGG	ATCATCATCCAGATCTCC
GAPDH	ACAGTTGCCATGTAGACC	TTTTTGGTTGAGCACAGG

Microarray analysis was performed by Phalanx Biotech Group (San Diego, CA). Total RNA was extracted from SR1423 and other B, C, D cell lines using RNA isolation kit (Qiagen) and RNA quantity and purity was assessed using NanoDrop ND-1000. RIN values are ascertained using Agilent RNA 6000 Nano assay to determine RNA integrity. Target preparation was performed using an Eberwine-based amplification method with Amino Allyl MessageAmp II aRNA Amplification Kit (Ambion, AM1753) to generate amino-allyl antisense RNA(aa-aRNA). Clustering was performed to visualize the correlations among the replicates and the differences between sample conditions. Up- and down-regulated genes are represented in red and green colors respectively. Data were analyzed by Rosetta Resolver^®^ System (Rosetta Biosoftware) and Generic pro software.

Cytogenetic analysis and DNA fingerprinting was performed by Cell Line Genetics (Madison, WI).

### Flow cytometry

Differentiated planar cultures of SR1432 were enzymatically released into single cells following incubation in Accumax (Stem Cell Technologies, #07921) at 37°C for 20–30 minutes. Cells were pelleted then resuspended in assay buffer (Millipore #CS202124) and quantified. Cells were fixed in 2% PFA for 10 minutes at 37°C, washed with assay buffer, then permeabilized in Methanol using PhosFlow Perm Buffer III (BD Biosciences #558050) for 30 minutes on ice. Cells were blocked in 1% Horse Serum in PBS for a minimum of one hour at 4°C. Cells were then apportioned into 100,000 cells per experimental sample and 500,000 cells for negative control. Anti-Pdx1 and anti-Nkx6.1 samples were incubated at 4°C overnight while anti-Insulin and anti-Glucagon samples were incubated for 30 minutes at room temperature. Cells were then washed in assay buffer twice and analyzed by a Millipore Guava 8HT flow cytometer. Data were subsequently analyzed with FlowJo software.

### Alginate encapsulation

Differentiated planar cultures of SR1423 were manually released using a cell lifter and rocked overnight at 95 RPM in a 6-well suspension culture dishes. Clusters formed were rinsed in 130 mM NaCl, 10mM MOPS, pH 7.4 and resuspended in 2% Pronova UP MVG alginate (Novamatrix) at a density of 2 x 10^6^ cells/ml. Alginate/cell mixture was loaded into a syringe and either fed through a Nisco electrostatic droplet generator at 4 mL/minute and 7kV with a 0.24 μm nozzle or manually dropped into a polymerization bath of 20 mM BaCl_2_, 130 mM NaCl, 10 mM MOPS. Beads were rinsed four times and returned to differentiation media until transplant.

### Animal studies

All animal work was performed as per the company’s animal care and use committee regulations. Induction of diabetes in mice: Immune-competent CD1 mice aged 8–10 weeks were used to induce diabetes with Streptozotocin (STZ, VWR # 102515–840). STZ was injected intraperitoneally (200 mg/kg) and STZ-induced diabetes was confirmed by blood glucose above 300 mg/dL for three consecutive days. Transplantation of alginate encapsulated pancreatic clusters: STZ induced diabetic mice were anesthetized with 20 mg/kg Tribromoethanol (Sigma # 776557888) and their abdomens shaved and sterilized with Isopropanol. A vertical incision was made in the middle of the abdomen below the sternum. Alginate beads were implanted to the peritoneum and the incision closed with sutures. Post-surgery, mice were given Ketoprofen (2.5 mg/kg, ThermoFisher #P08D009) for 3 days. Mice were observed regularly after transplantation. Blood glucose levels were monitored twice per week by taking a small drop of blood from the tail vein using a commercial glucometer.

### Ethics approval

SR1423, which was developed from a human pancreas and came from a consented anonymized deceased adult donor. His organs, including the pancreas, were re-covered by the California Transplant Donor Network in full compliance with U.S. ethics laws, after the completion of a donor medical and social history questionnaire. To confirm eligibility, the donor was screened for the presence of transmissible pathogens, according to 21 C.F.R. Part 1271 regulation.

All animal work was performed as per the company’s animal care and use committee regulations and received approval from Bioqual IACUC committee.

### Statistical analysis

All the data were from at least three independent experiments and checked the data for normal distribution. Expression level was analyzed by performing an unpaired two-tailed Student’s t test and two-way ANOVA followed by the Tukey-Kramer test. All statistical analyses were performed using the Prism7 (GraphPad Software). The differences were observed and considered statistically significant at the below 5% level and were displayed on the figures as follows: **P <0.05, ****p < 0.0001.

## Results

### Cell line derivation

**Fig 1:**
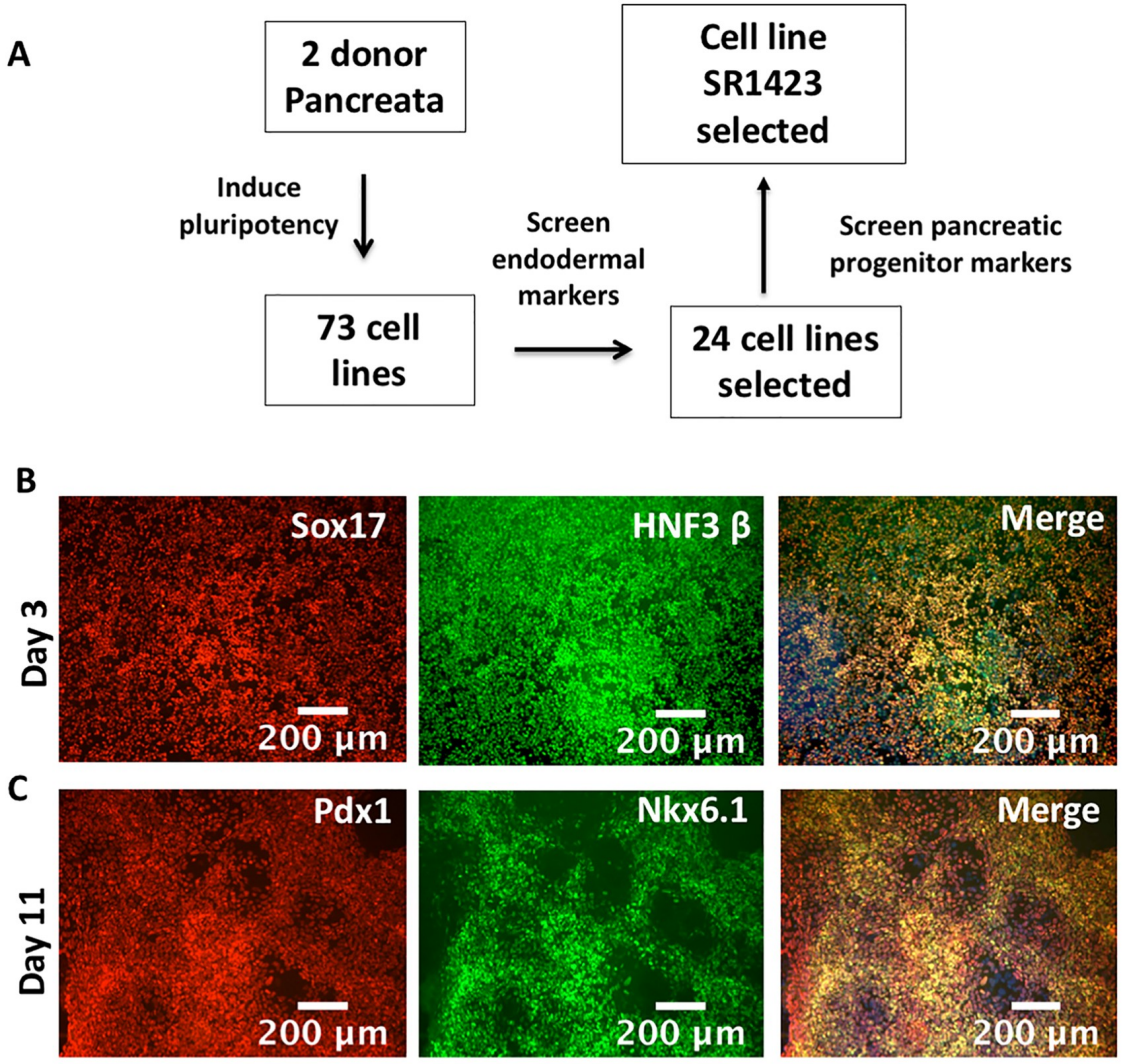
Immunostaining of endodermal and pancreatic progenitor cell markers in SR1423. Selection of iPSC line SR1423. (A) Selection scheme. (B) SR1423 expression of endoderm markers Sox17 (red) and HNF3ß (green) on Day 3 of differentiation. (C) SR1423 expression of pancreatic markers Pdx1 (red) and Nkx6.1 (green) on Day 11 of differentiation. “Merge” images include nuclear stain (blue). Scale bars are 100 or 200 μm, as indicated.

iPSC lines are generated by introducing reprogramming genes into the nuclei of mature cells. These genes, typically OCT4, SOX2, KLF4, and L-MYC can induce a subset of cells to adopt the gene expression pattern, morphology and behavior of embryonic stem cells [17]. It has been reported that epigenetic signatures of the starting cell population persist in reprogrammed beta cells [18], a phenomenon called “epigenetic memory”, although the duration of this effect is unknown [10]. To maximize the potential of generating an iPSC line that efficiently differentiates to the pancreatic lineage, we reprogrammed primary cells from the islets of Langerhans of consented healthy adult donor pancreata (Fig 1A).

**Fig 2:**
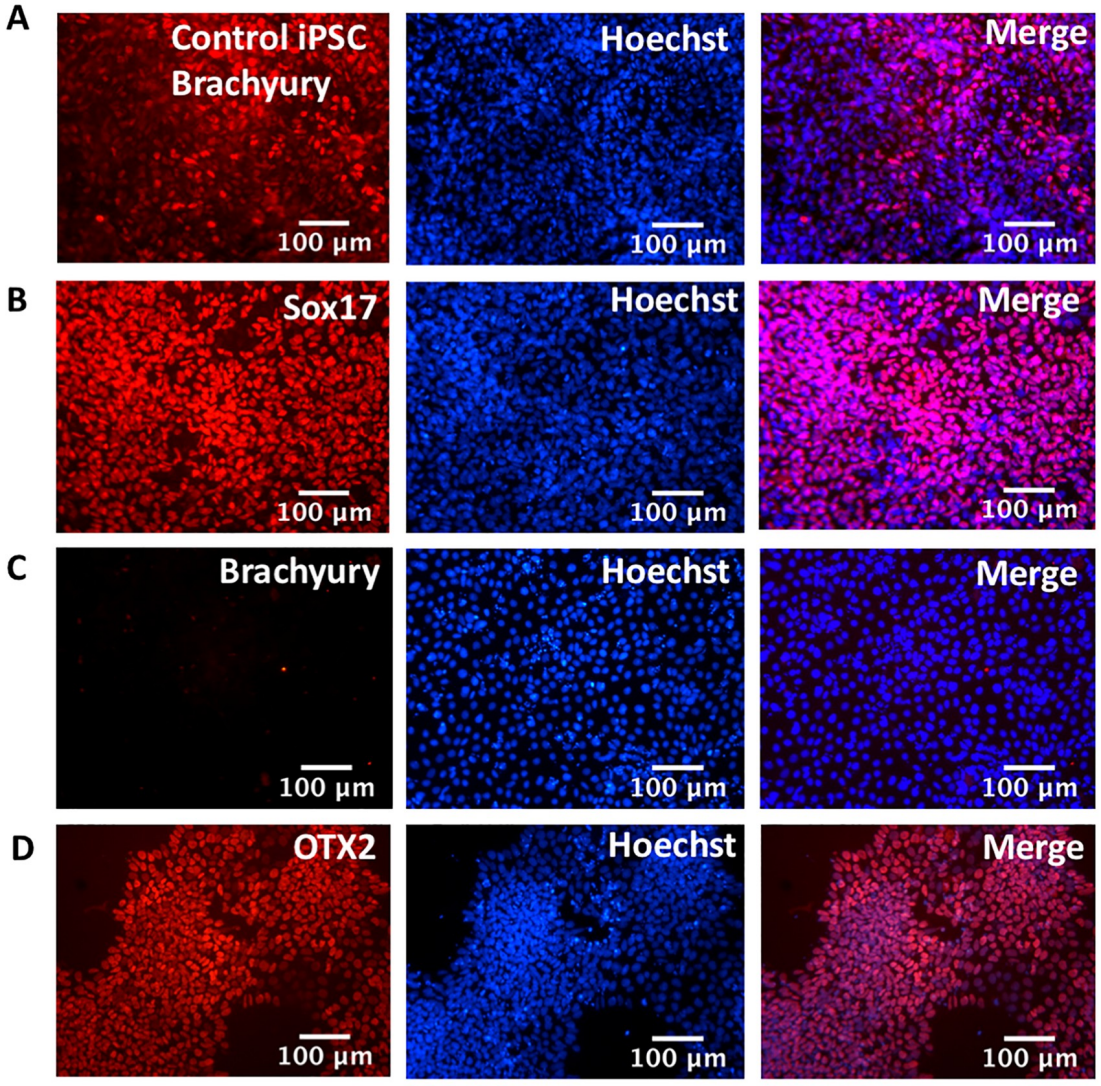
Expression of endoderm, mesoderm and ectoderm markers in immunoassayed SR 1423 cells. SR1423 cells differentiate poorly to the mesodermal lineage. (A) Positive control is an iPSC line that does not show a preference for endodermal differentiation. Differentiation of SR1423 into (B) Sox 17-expressing endoderm, (C) Brachyury-expressing mesoderm, and (D) OTX2-expressing cells ectoderm. Merged Images include nuclear stain (blue). Scale bar 100 μm. An example of three repeated experiments is shown.

Primary cells grown in cell culture can become homogenous and lose functional mature traits over time, possibly as a result of adaptation to artificial culture conditions [19]. To avoid the loss of genetic diversity in the starting cell population, we introduced reprogramming genes within five days of cell harvest. The reprogramming genes OCT4, SOX2, KLF4, and L-MYC were introduced to the primary cells via electroporation of two episomal expression plasmids. L-Myc was selected over C-Myc to reduce the potential of introducing an oncogenic gene [20]. Seventy-three lines generated from the primary tissue of two donors were initially screened for the ability to express endodermal markers after 4 days exposure to endoderm-inducing agents Activin-A and Wortmannin. Cultures with the highest proportion of cells expressing the endodermal markers Sox17 and HNF3beta were selected (S1 Fig). Twenty-four cell lines having passed the first screen were subsequently screened for the ability to express pancreatic markers after exposure to a 12-day pancreatic differentiation protocol. The cell line that consistently generated the highest proportion of pancreatic cells was named SR1423, was banked and used for all subsequent experiments (S2 Fig). This cell line generated nearly homogenous cultures of definitive endodermal (Fig 1B), and pancreatic progenitor cells (Fig 1C). Notably, SR1423 showed robust ability to differentiate to ectoderm and endoderm (as indicated by OTX2 and Sox17, respectively) but failed to express the mesodermal marker Brachyury when differentiated using a commercial kit (Fig 2). As all three germ layers were not attained, SR1423 does not fit the accepted criteria of pluripotency for iPSCs [21].

### Cell line characterization

**Fig 3:**
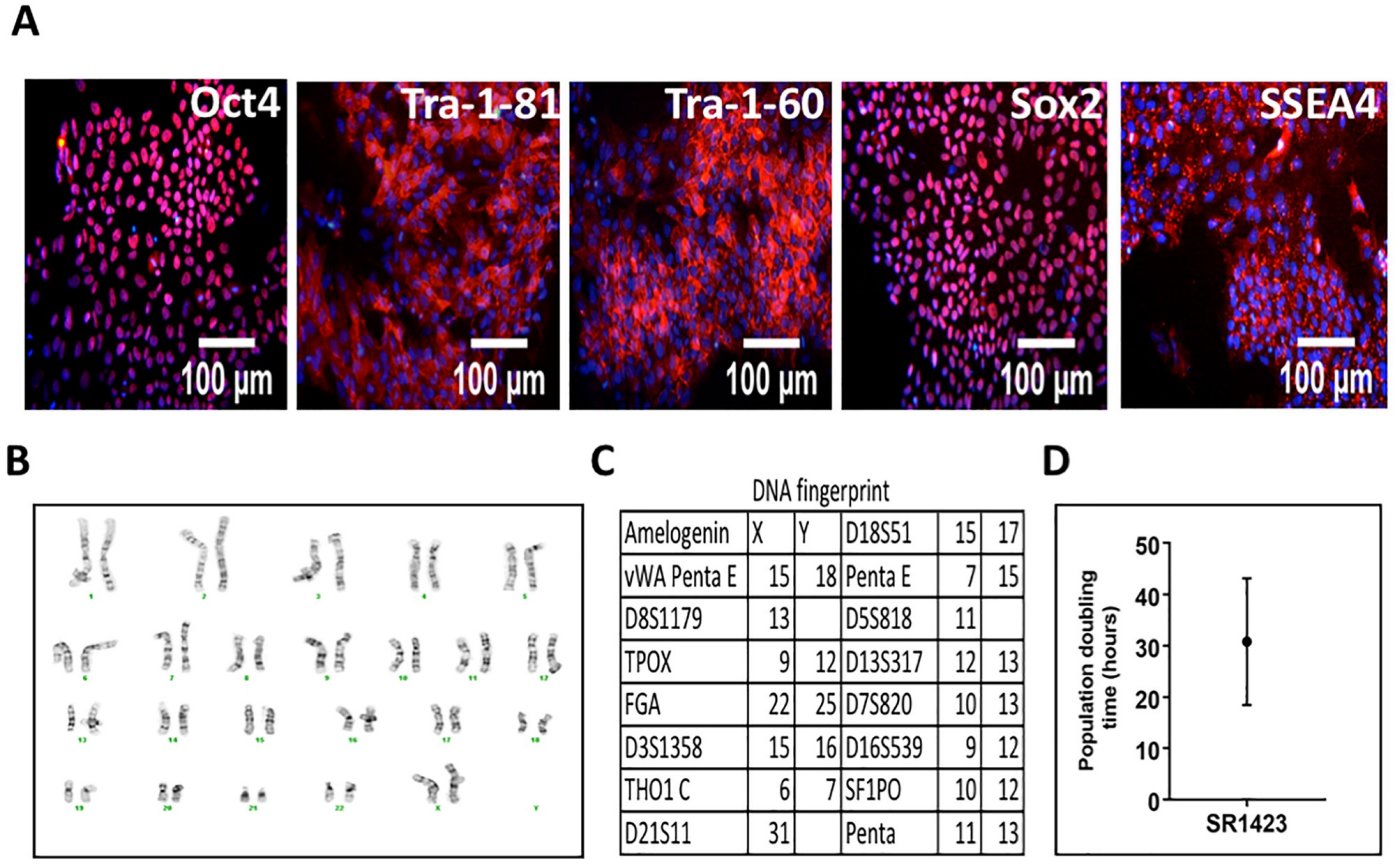
Characterization of SR1423. SR1423 cells have characteristics typical of pluripotent stem cells. (A) In the undifferentiated state, SR1423 expressed the markers of pluripotent stem cells Oct4, Tra-1-81, Tra-1-60, Sox2, and SSEA4. Images include nuclear stain (blue). (B) Cell karyotype after 40 passages in culture. Cells were karyotyped at passage 11 and passage 40 with identical results. (C) DNA fingerprint of SR1423 as assessed by single tandem repeat analysis (STR). (D) Column mean and error bar graph represents SR1423 cell doubling time. Scale bar 100 μm.

SR1423 expresses markers typical of pluripotent cells (Fig 3A) and has a normal karyotype (Fig 3B). Its DNA STR profile confirms a single cell line that matches the donor tissue (Fig 3C), and which is unique from all fingerprints in NIH, ATCC, and DSMZ databases. Additionally, SR1423 grows at a rate typical of pluripotent cell lines (Fig 3D).

**Fig 4:**
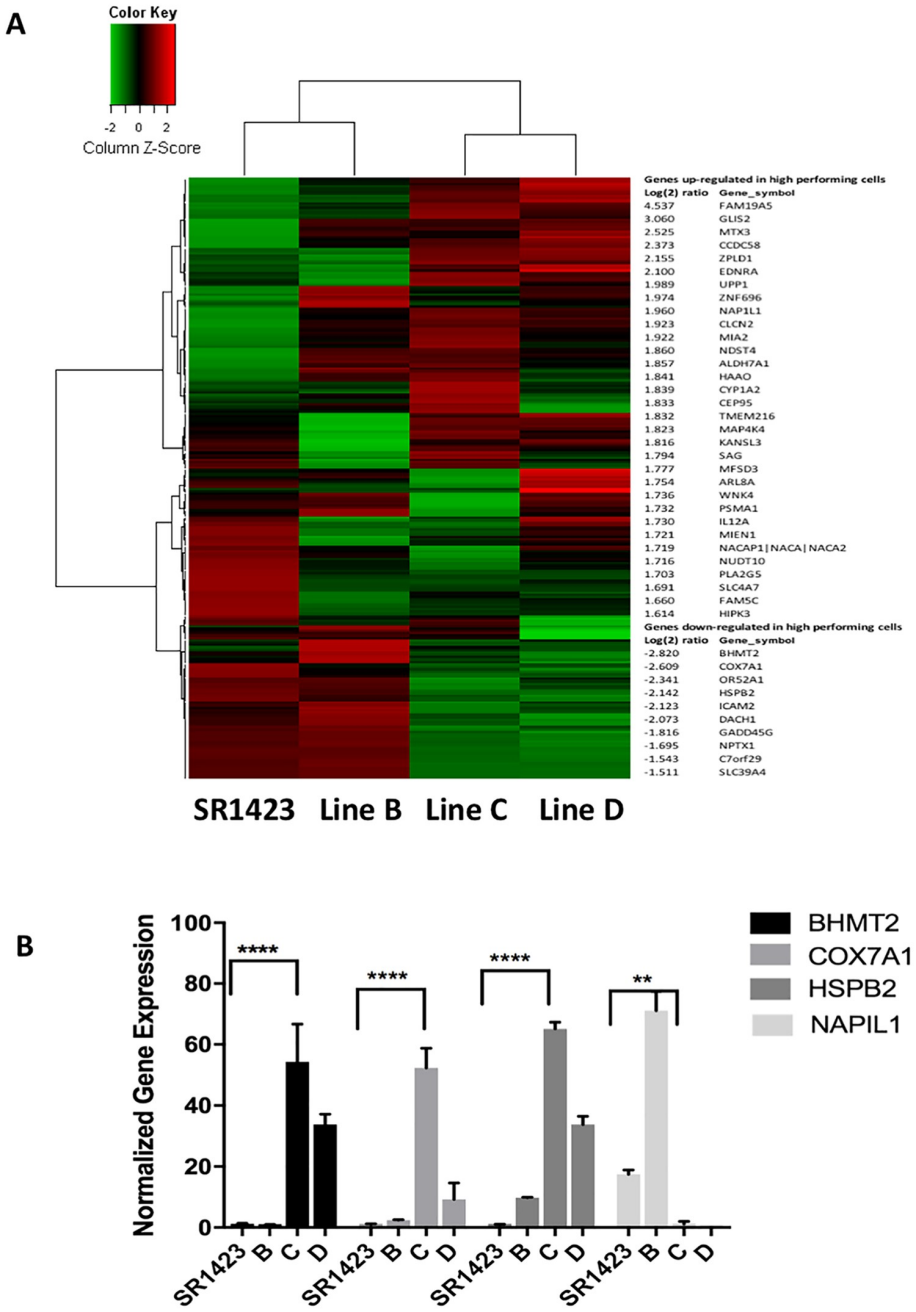
Whole- genome microarray and qRT-PCR analysis in SR1423. SR1423 gene expression pattern correlates with preferential differentiation to the endodermal lineage. (A) The correlation of expression profiles between two lines that demonstrate preferential differentiation to endoderm (SR1423 and B) and two lines that do not show preferential differentiation (C, D) was demonstrated by unsupervised hierarchical clustering analysis. Up- and down-regulated genes are represented in red and green colors, respectively. A subset of differentially expressed genes was selected from this clustering analysis based on an intensity filter that identifies genes with large expression differences between conditions. The 250 genes with the largest expression differences are represented. (B) qRT-PCR analysis of a selected subset of down- (BHMT2, COX7A1, HSPB2) and up-(NAP1L1) regulated genes identified in the gene expression analysis. Error bars represent the mean SD; **P<0.05; ****P<0.0001.

We observed that other iPSC cell lines from the same donor and reprogramming experiment demonstrated preferential differentiation to endoderm as well. To elucidate genes or pathways relevant to endodermal lineage preference, we performed whole-genome microarray profiling of expressed genes of SR1423 as well as alternative candidate cell lines B, C, and D. The iPSC line “B” also differentiated well to endoderm while iPSC lines “C” and “D” showed no preference for differentiation to the endodermal lineage (data not shown). By this comparison, differences in gene expression due to donor or methods of reprogramming are eliminated. Unsupervised hierarchical clustering analysis based on fold change expression of at least Log2, reveals that SR1423 clusters together with cell line B, but differently from C and D. This identifies a gene expression pattern that correlates with robust and preferential differentiation to the endodermal lineage (Fig 4A). Of the 10 most differentially expressed genes, BHMT2, COX7A1, HSPB2, and NAP1L1 correlated significantly with ability to form endoderm using a qRT-PCR measure (Fig 4B). Previous reports show correlation between gene expression profiles and the inability of iPSCs to differentiate into specific lineages [22]. These results raise the intriguing possibility that gene expression of a defined subset of genes could be used to identify a specific iPSC line with therapeutic utility and could subsequently be used as a quality control measure for cell therapy manufacturing to monitor genetic drift within cell cultures.

### Cell differentiation

Other groups reporting production of pancreatic cells from a pluripotent stem cell population use a unique cell culture protocol in combination with a unique stem cell population [8,9,23–27]. Each of these protocols was tailored for a particular starting cell population, further lending credence to the concept that the starting cell population is a main determinant of differentiation potential. However, there are areas of homology across protocols.

Generation of beta cells occurs by the progressive differentiation of the pluripotent cells through the known stages of embryonic pancreatic development [6]. This progression begins with formation of definitive endoderm, followed by transition to pancreatic progenitor, endocrine-committed pancreas, and finally, hormone-expressing pancreatic cells. Production of definitive endoderm cells expressing Sox17 and HNF3beta is accomplished by exposure to Activin A and Wnt3a, signaling molecules involved in endodermal patterning in mammals [28,29]. Pancreatic progenitors, identified by expression of pancreatic duodenal homeobox-1 (Pdx1), arise after activation of HOX genes with retinoic acid, while inhibiting hedgehog signaling with cyclopamine [30]. Endocrine cells expressing both Pdx1 and Nkx6.1 are formed from pancreatic progenitors by activation of KGF signaling, involved in the formation of pancreatic duct cells, in the presence of the patterning protein noggin [31]. Maturation to the hormone expressing phenotype is reported to be encouraged by thyroid hormone [32]. Significant effort has been made to replace growth factors and hormones employed in differentiation protocols with small molecules [33–35].

**Fig 5:**
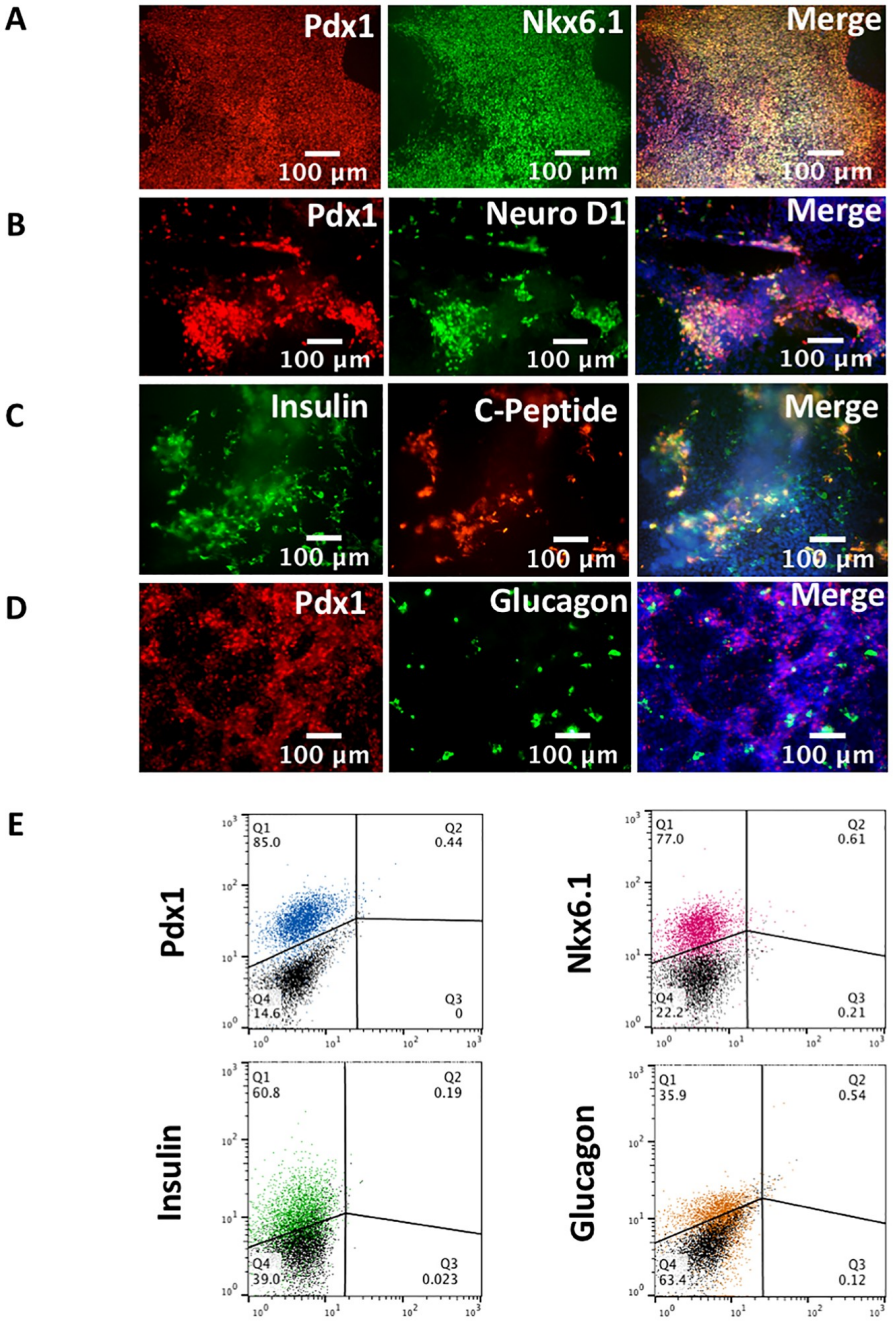
Characterization of SR1423 by endocrine pancreatic markers expression and quantification. SR1423 differentiation yields robust pancreatic, hormone-secreting cell populations. (A) SR1423 differentiation labeled for Pdx1 (red) and Nkx6.1 (green) expression. (B) SR1423 differentiation labeled by Pdx1 (red) and NeuroD1 (green). (C) SR1423 differentiation labeled for Insulin (green) and C-peptide (red). (D) SR1423 differentiation labeled for Pdx1 (red) and Glucagon (green). “Merge” images include nuclear stain (blue). All images were taken after 28 days of differentiation under 40X original magnification (n = 10). Scale bar 100 μm. (E) Flow cytometry of SR1423 differentiated for 24 days. Cells stained with anti-Insulin (green), anti-Pdx1 (blue), anti-Nkx6.1 (red), and anti-Glucagon (orange) displayed with overlays of negative controls (black) in each panel. Quadrants are labeled with percentages of experimental cells within the spider-gated region (n = 5000 cells). An example of three repeated experiments is shown.

We formulated and optimized a protocol incorporating these elements to drive the differentiation of SR1423 to the beta cell phenotype. Our protocol generated highly pure populations of endocrine pancreatic cells expressing the Pdx1 (Fig 5A, red), Nkx6.1 (Fig 5A, green) and NeuroD1 (Fig 5B, green) markers by day 28 of differentiation. Insulin expression was also detected by direct staining as well as by C-peptide expression (Fig 5C, green and red respectively). Additionally the hormone glucagon was expressed in a small population of cells as well (Fig 5D, green).

Due to the 3-dimensional nature of the differentiated planar culture, the true extent of expression is difficult to glean from images. Quantification by flow cytometry revealed populations comprised of cells in which 60.8% of cells were positive for insulin expression (Fig 5E), while the endocrine pancreatic markers Pdx1 and Nkx6.1 were expressed in 85% and 77% of cells, respectively (Fig 5E). We also assayed for expression of glucagon (Fig 5D, quantified in Fig 5E) and found that approximately 36% of cells were positive. To determine if this result was specific to our reprogrammed cell lines, we differentiated a reference embryonic stem cell line BGO1V following our protocol. Our protocol also generated mature, pancreatic cells using this line, though less efficiently (Supplemental S3 Fig).

**Fig 6:**
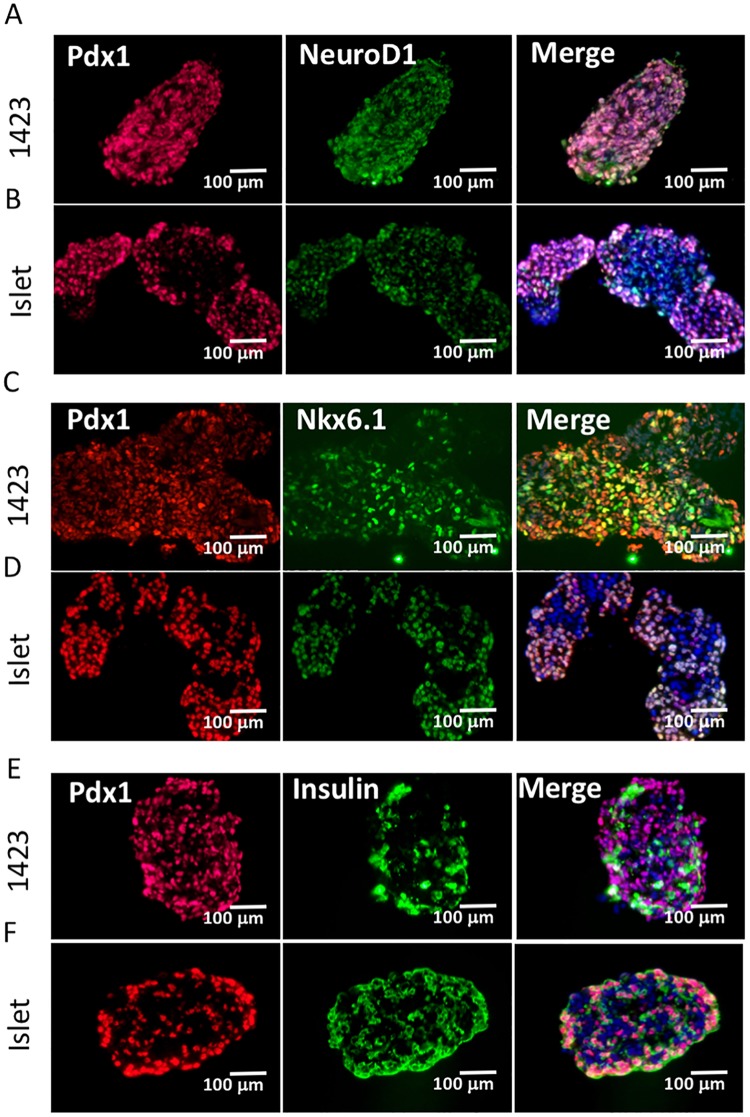
Characterization of SR1423 differentiated cell clusters compared to human islets. (A) Immunofluorescence of SR1423 differentiated cell clusters (A, C, E) and human islets (B, D, F) expressing Pdx1 (A-E, red), NeuroD1 (A, B green), Nkx6.1 (C, D green), and Insulin (E, F green). Scale bar 100 μm.

To demonstrate how our cultures compare to a human islet, expression of Pdx1 and Nkx6.1, and of Pdx1 and insulin are shown in (Fig 6A–6F). To generate a clear comparison, the adherent cultures were transitioned to suspension culture as described in the Materials and Methods. While size of clusters are much more variable than islets, these examples demonstrate that expression of Pdx1, NeuroD1, and Nkx6.1 in SR1423 cell clusters (Fig 6A and 6C) is similar in abundance and distribution to that of human islets (Fig 6B and 6D). The arrangement of insulin-expressing cells in SR1423 differentiated clusters also appeared similar to that of human islets (Fig 6E and 6F).

**Fig 7:**
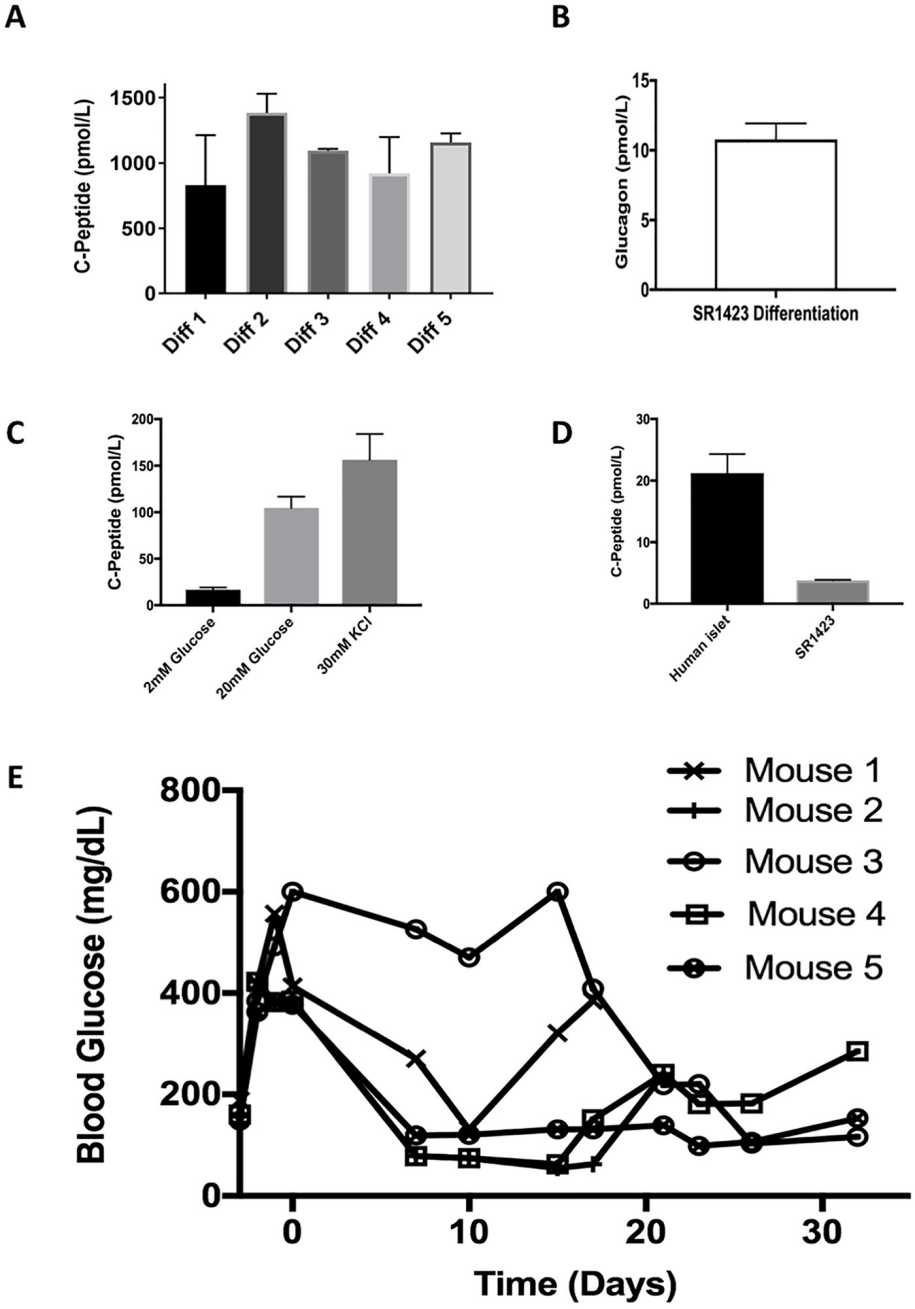
Immunoassay of C-Peptide and Glucagon in SR1423 beta-like cell cultures. SR1423 differentiation yields cultures with high levels of hormone-secretion. Insulin and Glucagon levels were assessed via C-peptide (as a proxy for Insulin) or Glucagon ELISA. (A) C-peptide ELISA over 5 consecutive differentiations. Assay performed on planar cell cultures. (B) Glucagon secretion in response to membrane depolarization (30mM KCl). (C) Glucose responsiveness of C-peptide levels in islet-like cell clusters at 2mM and 20mM glucose and 30mM KCl (*n* = 3) (D) C-peptide secretion upon membrane depolarization (30mM KCl) per pdx-expressing islet-like cell cluster or human islet. E) Blood glucose values of 5 mice treated with 25,000–40,000 cells per gram of body weight of differentiated SR1423 in STZ-treated mice.

We also observed that cells differentiated from SR1423 following our protocol secrete insulin (Fig 7A) and glucagon (Fig 7B) into the media. Sequential differentiations of SR1423 show consistent, reproducibly high levels of C-Peptide detection (Fig 7A) from planar cultures. For comparability, planar cultures were broken into cell clusters similar to islets. C-peptide secretion was then measured in low (2mM) and high glucose (20mM) and upon membrane depolarization with 30mM KCl (Fig 7C). To compare the amount of insulin available for secretion of SR1423 to that of human islets, the amount of C-peptide secreted during a static incubation in high glucose and KCl was divided by the number of Pdx1-expressing cell clusters in each population (Fig 7D). This analysis reveals that SR1423 clusters secreted 17% of the C-peptide secreted by equivalent numbers of human islets. Consequently, these hormone-secreting cells may be ideal candidates for cell replacement therapies.

### Reversal of diabetes in an animal model

A common method for immune-protecting islet cells for transplant is to embed the cells within alginate-containing microbeads [36]. Surrogate pancreatic cells embedded within alginate and implanted to the peritoneum can demonstrate short-term reversal of diabetes and provides a good basis for a proof of concept. Microbeads formed of modified alginate with a lower tendency to stimulate fibrosis was able to reverse diabetes in normal rodents for up to 6 months [5]. These authors also reported that larger microbeads were resistant to the accumulation of fibroses that would otherwise form a barrier around the alginate beads [37]. The accumulation of fibroses can be further inhibited in a macro-capsule design with the use of ultra-pure alginates with high guluronate content [38]. To demonstrate the ability of SR1423-generated cells to reverse diabetes within an immune-protective device, we embedded the differentiated cells within alginate macro-capsules employing ultra-pure alginate with high guluronate content and implanted these to the peritoneum of normal mice with chemically induced diabetes. Doses above 25,000 cells per gram of body weight consistently lowered blood glucose. Lower blood glucose was evident within 7–21 days of transplant and was maintained for a period of weeks (Fig 7E).

## Discussion

In this study, we derived SR1423 specifically for the purpose of creating a cell line that would be useful as a diabetes cell therapy. It was anticipated that a cell line chosen for its ability to differentiate to the pancreatic lineage might form significantly higher numbers of insulin-expressing cells. Indeed, SR1423 was able to generate up to 60.8% insulin-expressing cells after 28 days in adhesion culture. Despite initiating differentiation as planar culture, many 3D structures occur spontaneously during maturation. Other protocols for equivalent beta cell differentiation have employed suspension culture [8,39], growth in a liquid-air interface [9], or differentiation in vivo [40,41] to achieve insulin secretion. Though it is unknown whether the ability to differentiate well in adhesion culture is a trait specific to SR1423, our data suggests that our simpler protocol was able to robustly generate mature pancreatic cells from reference hESC line BGO1V in adhesion as well.

While protocols for generation of insulin-producing cells in vitro vary depending on the cell line, many common themes are found among them [8,9,14,15]. Two such commonalities are the inhibition of the activin receptor-like kinase (Alk5i) and employing a high concentration of the hormone tri-iodothyronine (T3). While optimizing our protocol with SR1423, we found that both molecules are dispensable. Alk5i is an inhibitor of TGFbeta signaling. Evidence suggests that TGFbeta may participate in signaling activated by Hedgehog [42]. Hedgehog signal inhibition is targeted in the protocol with application of cyclopamine, which may render Alk5 inhibition redundant. Likewise, application of nicotinamide depletes the thyroid hormone receptor of rat pituitary cells and activates the rat growth hormone gene promoter [43,44]. It is possible, therefore that nicotinamide and its main target Insulin-like growth factor-1 (IGF-1), can have overlapping function with T3 in the context of the developing beta cell. Both nicotinamide and IGF-1 are employed in the protocol reported here.

We found that extensive screening was required to identify pluripotent cell lines capable of achieving beta cell identity, despite having been derived from islet cells. Seventy-four pluripotent cell lines were derived from the donor islets, yet only 24 lines were capable of differentiating to endoderm efficiently (Fig 1A). Further, only two cell lines robustly expressed markers of pancreatic progenitor fate following differentiation. Reports of epigenetic memory in beta cell derived iPSC [18] do not mention assessing iPSC line quality, yet we found screening to be absolutely necessary to achieve cultures that generated high numbers of beta cells. It is possible, but not explored here, that the characteristics of the donor tissue influenced the range of genetic diversity in the resulting iPSC line. The organ donor, deceased as a result of head trauma, was a Hispanic male aged 25, non-obese and non-diabetic and with no other known medical conditions. In this case, we reprogrammed endodermal tissue to generate an iPSC that preferentially differentiated back to the endodermal lineage. Likely, this strategy could also generate iPSC lines with preferential differentiation to the mesodermal and ectodermal lineages when starting with mesodermal and ectodermal primary cells.

Our screening methods identified a pluripotent cell line with an endodermal inclination that readily differentiates to pancreatic fate. We found by gene analysis that these cells have a unique profile compared to those that had no preference for endoderm. The four genes that correlated with endodermal inclination by qRT-PCR analysis were largely expressed non-specifically. However, BHMT2 [45] and NAP1L1 [46,47] both have potential roles in DNA modification and may contribute to epigenetic memory. In support of this idea, Nap1L1 knock-down has been reported to accelerate mesoderm formation in murine iPSCs [48]. Determining if these proteins are involved in the patterning for differentiation preference among pluripotent cells will be key for generating predictive criteria.

ICR/CD1 mouse strain, one of the most widely used rodents for biomedical research, was chosen for this study for being a stringent model for transplant and diabetes studies [49,50]. It is hypothesized that outbred mouse strains, due to their greater genetic diversity, better model cell-mediated transplant rejection responses than inbred mice [49]. Also, it has been reported that inbred mouse strains vary in their tendency to form fibroses after injury or around foreign object implants [51,52]. The formation of fibroses are a form of chronic rejection that will lead to long-term failure of the implant by choking off the implanted cells from the host blood supply and is therefore a relevant parameter in the choice of mouse strain. The outbred CD1 mouse is shown to be susceptible to fibrotic deposition [53,54]. Finally, the CD1 mice respond similarly to human insulin as other common mouse strains [50].

In anticipation of beginning clinical studies, SR1423 is being assessed in toxicology, tumorigenicity, and biodistribution assays pursuant to pre-IND interactions with the FDA. Preliminary studies to be presented in a subsequent article suggest that a facile ability to differentiate may also lead to a low proportion of residual undifferentiated cells in the therapeutic population, and a low tumorigenic potential. It is known that pluripotent stem cells can potently form teratomas but that iPS cells can have varying tumorigenic potential [55]. Our strategy not only has an advantage in terms of an increase in the proportion of insulin-producing cells generated, but also a low proportion of residual undifferentiated cells leading to a low tumorigenic potential.

For a practical therapy for diabetes, a strategy to enable cell survival and function in the host in the absence of systemic immune suppression is essential. Here we used alginate encapsulation followed by implant to the peritoneum. While this system was able to restore normoglycemia for several weeks, previous studies have demonstrated that such implants do not function long-term [56]. Significant effort will be needed to develop an encapsulation system that is biocompatible, does not stimulate inflammation or fibroses, enables vascularization, and can be retrieved.

## Conclusions

Reprogramming and selection of a cell line for a diabetes cell therapy successfully generated a cell line with a differentiation preference towards endodermal tissue. Coupled with an optimal protocol, the cell line consistently created highly pure, insulin-producing cells capable of reversing diabetes in animal models.

## Supporting information

S1 Fig Differential expression of Sox17 and HNF3beta in candidate iPSC lines. Selected line SR1423 (A) and an unselected iPSC line (B) expression of Sox17 (red) and HNF3beta (green) on day 4 of differentiation. “Merge” images include nuclear stain (blue). Scale bar 200 μm.
(TIFF)

S2 Fig Differential expression of Pdx1 and Nkx6.1 in candidate iPSC lines. Selected line SR1423 (A) and two unselected iPSC lines (B, C) expression of Pdx1 (red) and Nkx6.1 (green) on day 13 of differentiation. “Merge” images include nuclear stain (blue). Scale bar 200 μm.
(TIFF)

S3 Fig Quantification of BGO1V expression of pancreatic markers. Flow cytometry of BGO1V differentiated for 28 days. Cells stained with anti-Pdx1 (blue) and anti-Nkx6.1 (red) displayed with overlays of negative controls (black) in each panel. Quadrants are labeled with percentages of experimental cells within the spider-gated region (n = 5000 cells). An example of 3 repeated experiments is shown.
(TIFF)

## Abbreviations

ATCC: American Type Culture Collection
DSMZ: Deutsche Sammlung von Mikroorganismen und Zellkulturen
hESC: human embryonic stem cells
iPSC: induced pluripotent stem cell
NIH: National Institutes of Health
qRT-PCR: quantitative real-time PCR
