# Correction: Generation and selection of pluripotent stem cells for robust differentiation to insulin-secreting cells capable of reversing diabetes in rodents

**DOI:** 10.1371/journal.pone.0224944

**Published:** 2019-10-31

**Authors:** Sheryl M. Southard, Rama P. Kotipatruni, William L. Rust

There are a number of errors in Fig 3, “Characterization of SR1423,” panels B-C. Please see the complete, correct Fig 3 here.

**Fig 3:**
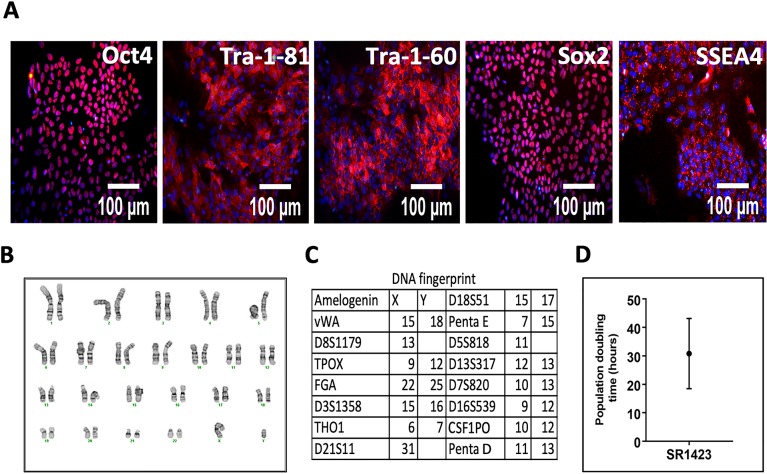
Characterization of SR1423. SR1423 cells have characteristics typical of pluripotent stem cells. (A) In the undifferentiated state, SR1423 expressed the markers of pluripotent stem cells Oct4, Tra-1-81, Tra-1-60, Sox2, and SSEA4. Images include nuclear stain (blue). (B) Cell karyotype after 40 passages in culture. Cells were karyotyped at passage 11 and passage 40 with identical results. (C) DNA fingerprint of SR1423 as assessed by single tandem repeat analysis (STR). (D) Column mean and error bar graph represents SR1423 cell doubling time. Scale bar 100 μm.
