# Supplementary figures and images for: Generation and selection of pluripotent stem cells for robust differentiation to insulin-secreting cells capable of reversing diabetes in rodents
Source: PLoS One. 2018 Sep 5;13(9):e0203126. doi: 10.1371/journal.pone.0203126 (PMC6124757; doi:10.1371/journal.pone.0203126)

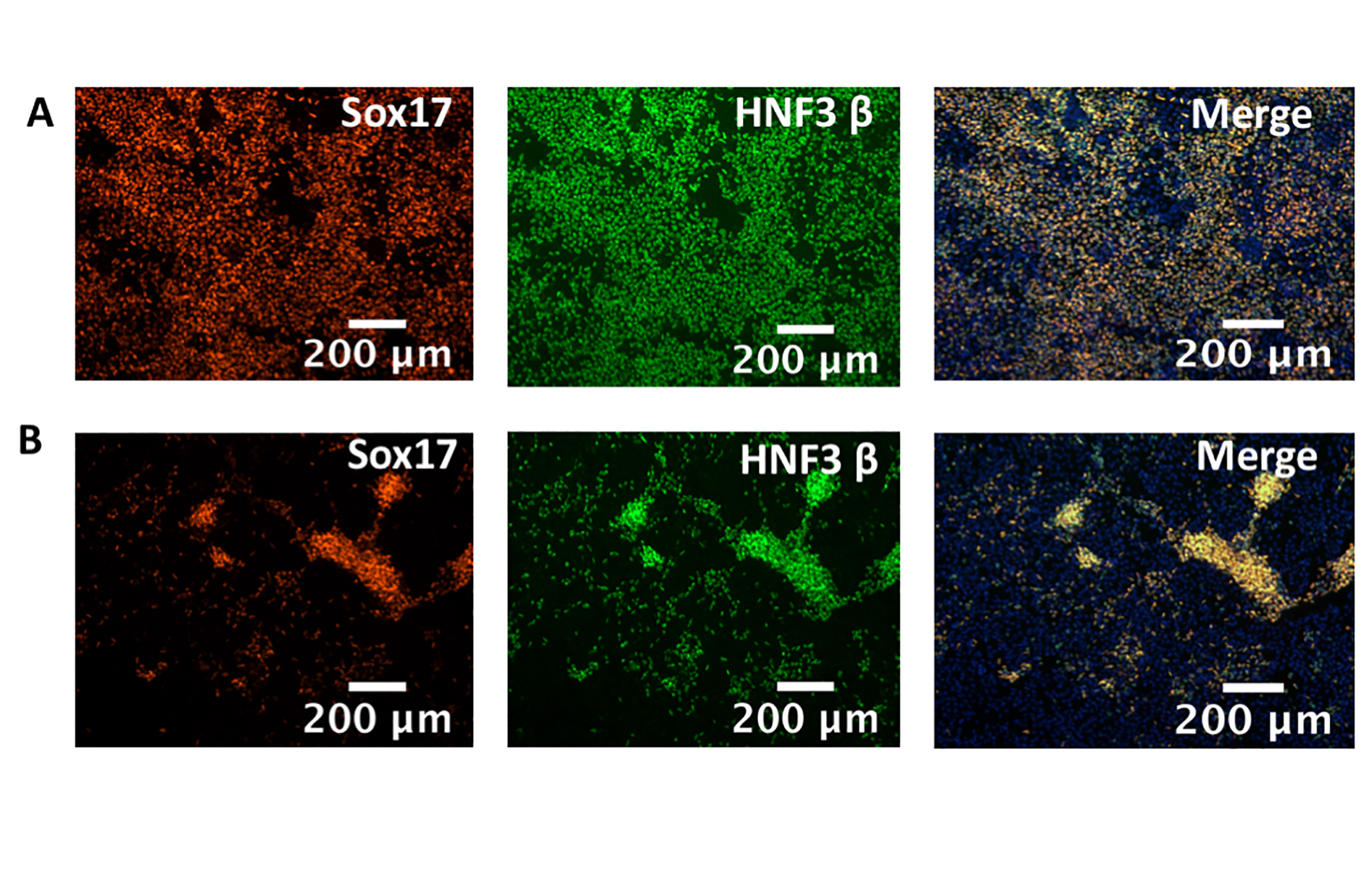

Supplement: S1 Fig — Selected line SR1423 (A) and an unselected iPSC line (B) expression of Sox17 (red) and HNF3beta (green) on day 4 of differentiation. “Merge” images include nuclear stain (blue). Scale bar 200 μm. (TIFF) [file pone.0203126.s001.tiff]

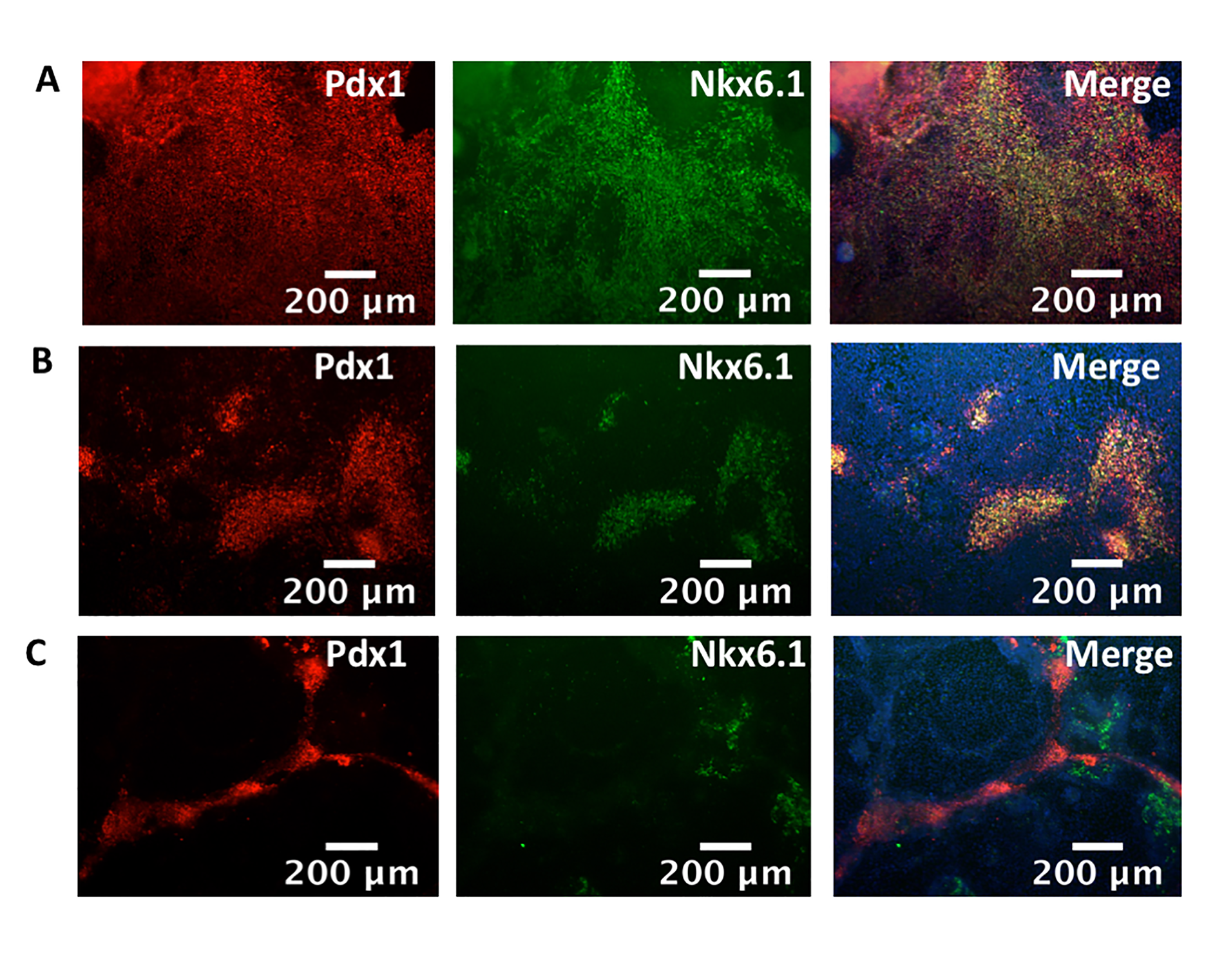

Supplement: S2 Fig — Selected line SR1423 (A) and two unselected iPSC lines (B, C) expression of Pdx1 (red) and Nkx6.1 (green) on day 13 of differentiation. “Merge” images include nuclear stain (blue). Scale bar 200 μm. (TIFF) [file pone.0203126.s002.tiff]

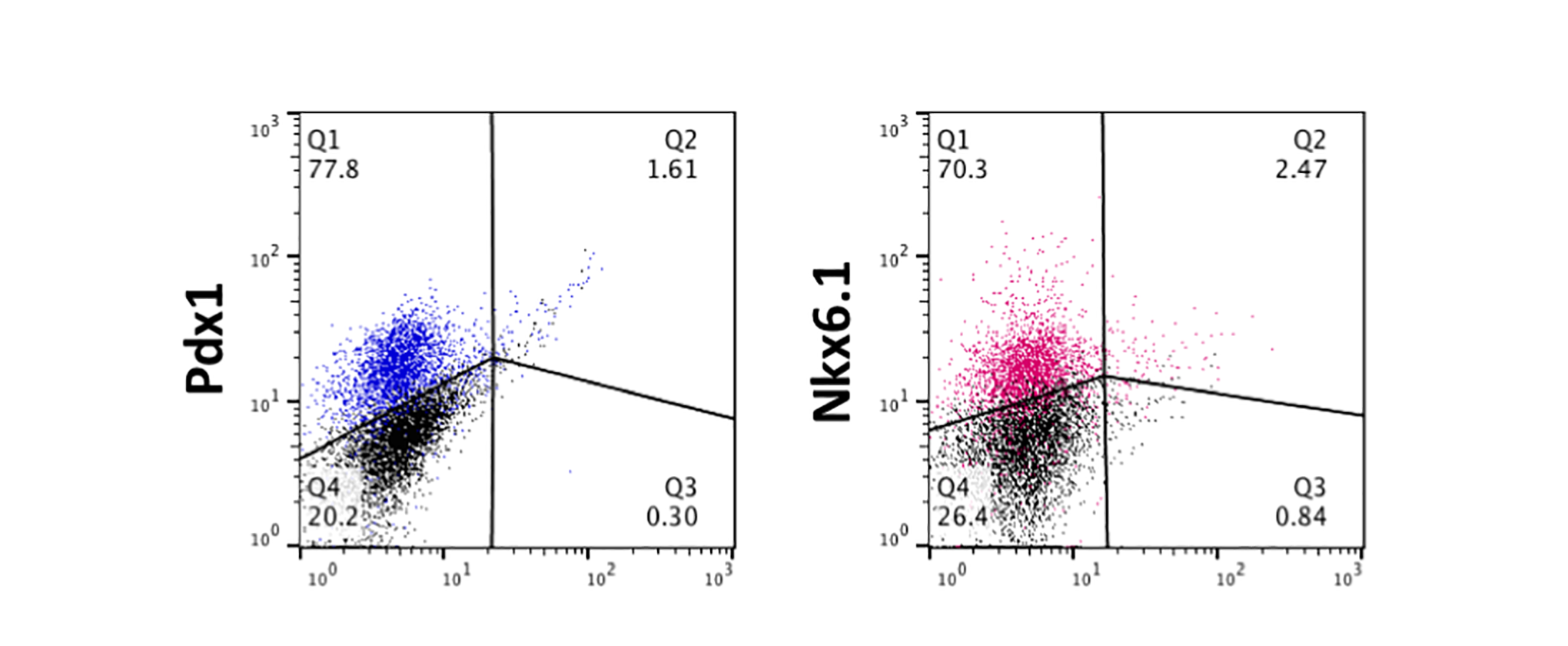

Supplement: S3 Fig — Flow cytometry of BGO1V differentiated for 28 days. Cells stained with anti-Pdx1 (blue) and anti-Nkx6.1 (red) displayed with overlays of negative controls (black) in each panel. Quadrants are labeled with percentages of experimental cells within the spider-gated region (n = 5000 cells). An example of 3 repeated experiments is shown. (TIFF) [file pone.0203126.s003.tiff]
